# Investigation of mid-infrared emission characteristics and energy transfer dynamics in Er^3+^ doped oxyfluoride tellurite glass

**DOI:** 10.1038/srep10676

**Published:** 2015-06-02

**Authors:** Fangze Chen, Tao Wei, Xufeng Jing, Ying Tian, Junjie Zhang, Shiqing Xu

**Affiliations:** 1 College of Materials Science and Engineering, China Jiliang University, Hangzhou 310018, PR China; 2 Institute of Optoelectronic Technology, China Jiliang University, Hangzhou 310018, PR China

## Abstract

Er^3+^ doped oxyfluoride tellurite glasses have been prepared. Three Judd-Ofelt parameters Ω_t_ (t = 2, 4, 6) and radiative properties are calculated for prepared glasses. Emission characteristics are analyzed and it is found that prepared glasses possess larger calculated predicted spontaneous transition probability (39.97 s^−1^), emission cross section σ_em_ (10.18 × 10^−21^ cm^2^) and σ_em_ × Δλ_eff_ (945.32 × 10^−28^ cm^3^), corresponding to the 2.7 μm emission of Er^3+^: ^4^I_11/2_→ ^4^I_13/2_ transition. The results suggest that the prepared glasses might be appropriate optical material for mid-infrared laser application. Moreover, rate equation analysis which is rarely used in bulk glass has been carried out to explain the relationship between emission intensity and Er^3+^ concentration. The calculation results show that with the increment of Er^3+^ concentration, the energy transfer up-conversion rate of ^4^I_13/2_ state increases while the rate of ^4^I_11/2_ state reduces, resulting in the change of 2.7 μm emission.

Nowadays a significant increment of interest has been shown in the Er^3+^ doped materials. Reasons could be explained that the 2.70 μm emission generated by Er^3+^: ^4^I_11/2_ → ^4^I_13/2_ transition has numerous applications in military weapons, remote sensing, and laser surgery^1^,^2^. Moreover, transitions of ^4^I_11/2_ → ^4^I_15/2_ and ^4^I_9/2_ → ^4^I_15/2_ can be easily pumped by 980 nm and 808 nm commercial laser diode (LD), respectively. As is known, pumping at 980 nm directly into the upper ^4^I_11/2_ state results in the highest efficiency of 35%^3^. However, the existed excited-state absorption transition (ESA) of ^4^I_11/2_ → ^4^F_7/2_ that depletes the population in ^4^I_11/2_ state is unfavorable for 2.7 μm emission and must be avoided^4^. Experimentally, the best pump wavelength is near 792 nm because this wavelength is at the peak of cascade ESA transition of ^4^I_13/2_ → ^2^H_11/2_, which brings about a redistribution of population in ^4^I_13/2_ state and benefits the 2.7 μm emission^5^.

It is widely recognized that ^4^I_11/2_ → ^4^I_13/2_ transition of Er^3+^ is a self-terminating process, which means the upper ^4^I_11/2_ state has a short lifetime than the lower ^4^I_13/2_ state. Consequently, it requires some ways to deplete the lower state population and conserve upper state population for efficient 2.7 μm laser operation. Efficient approaches for population depletion consist of co-lasing of Er^3+^: ^4^I_13/2_ → ^4^I_15/2_ + 1.53 μm photon, energy transfer to sensitizing ions and energy transfer up-conversion: ^4^I_13/2_ +  ^4^I_13/_ → ^4^I_15/2_ + ^4^I_9/2_ (ETU1) at high Er^3+^ concentration^6^. Moreover, the energy transfer up-conversion of ^4^I_11/2_ + ^4^I_11/2_ → ^4^F_7/2_ + ^4^I_15/2_ (ETU2) is harmful for conserve upper state population. The first two ways have been widely investigated according to various reports. However, as far as we known, few quantitative analysis of energy transfer up-conversion has been investigated in bulk glasses. By building proper equations, it is possible to quantitatively analyze energy transfer mechanism, which is one of major work we made in following paper.

In addition, host materials with low phonon energy that decreases the non-radiative relaxation rate are desirable for 2.7 μm laser operation. Therefore, it is crucially important to find a host material, which is suitable for both optical and physical requirement in order to obtain outstanding 2.7 μm laser properties. Recent reports have witnessed the development of various host glasses such as fluoride^7^, fluorophosphate^8^, germanate^9^ and chalcogenide^10^. The highest output power achieved in Er^3+^ doped fluoride glass fiber has been reported by S. Tokita in 2011. With the help of liquid-fluorocarbon that dissipates the heat, the output power reaches up to 24 W with slope efficiency of 14.5%. Since then, new progress of higher output in Er^3+^ doped fluoride glass fiber has hardly been reported. Except for fluoride glass, the mid-infrared laser output of these following glasses has not yet achieved up to now. The fluorophosphate glass, which combines the merits of fluoride glass and phosphate glass, does not show the good thermal stability and low phonon density as expected. Many efforts should still be made for practical use. The industrial development of germanate glass for mid-infrared laser is blocked by the drawbacks of high viscosity, high melting temperature and large amount of hydroxyl groups^11^. Although mid-infrared laser output of Pr^3+^ doped chalcogenide glass has been reported, due to its cost and fragility, significant efforts are still in the way to realize high output power in this kind of fiber laser for practical applications^12^,^13^.

As a conditional glass network former, tellurium dioxide (TeO_2_) has low phonon energy (~760 cm^−1^) and large refractive index, both of which are beneficial for high radiative index values. Moreover, the tellurite glasses show good thermal property and high doping concentration of rare earth ions without clustering, which make them a good luminescence material. However, the tellurite glass also has the problem of hydroxyl groups and tellurite glass fiber with low-loss is hard to be prepared due to the inhomogeneity of tellurite glass, which prevents them to be widely applied as mid-infrared material^14^. Oxyfluoride tellurite glasses (OFT), added with certain amount of fluoride, combine the advantages of oxide glasses with the good optical properties of fluorides. The addition of fluorides, on the one hand, could further decrease the phonon energy of glass matrix; on the other hand, it is an effective way to exhaust OH^−^ content due to the reaction of OH^−^ + F^−^ → O^2−^ + HF≠. The two main categories of OFT glasses are TeO_2_-ZnO-Na_2_O (TZN) based and TeO_2_-WO-La_3_O_2_ (TWL) based systems. The TWL system has very good thermal stability due to the existence of W-O banding in network. There is no obvious crystallization temperature (T_x_) in some TWL glasses; therefore, it is quite beneficial for fiber drawing. However, the W-O banding located at 930 cm^−1^ in Raman spectra leads to high phonon energy^15^. The other challenge for TWL system is the difficulty of introducing F^−^ ions into the glass matrix. Thus, the TWL system based OFT glass with large amount of fluoride has not reported yet. On the contrary, the TZN based system has quite low phonon energy (mainly due to the vibration of Te-O banding at 760 cm^−1^) and is easy to bring F ions in the glass matrix. The water-free OFT glass fiber based on TZN system has been achieved and shows good property^16^. However, the transmittance of it reaches around 80% and the glass transition temperature (T_g_) of it is only 280 °C. Both the two factors are disadvantageous to high power and high optical performance laser material.

In this research, a kind of OFT glasses with molar compositions of 50TeO_2_ - 39RF_2_ (R = Ba, Mg, Zn) - 3NaF - 8 YF_3_ - xErF_3_ (x = 1, 3, 5, 7, 9) is reported. The large amount of TeO_2_ is the main glass network former and provides the main thermal and optical properties. The bivalent alkaline-earth ions were introduced into the matrix to enhance the emission of rare earth ions and play as the glass network modifier^17^,^18^. The introduction of large amount of F^−^ ions decreases the phonon energy and depletes the OH^−^. The addition of YF_3_ into the matrix increases the viscosity of the melt and finally enhances the stability of the glass against crystallization^19^.Judd-Ofelt intensity parameters are determined to calculate the radiative transition probability and radiative lifetime of excited states. The 1.53 μm, 2.7 μm and 980 nm emissions under 808 nm excitation are investigated. Additionally, the rate equation analysis is used to calculate ^4^I_13/2_ state and ^4^I_11/2_ state energy transfer up-conversion rates as a quantitative explanation for energy transfer dynamics. In our previous work^11^,^20^, compositional dependence of energy transfer up-conversion rate has been investigated. In this work, we further investigate the Er^3+^ concentration dependence of energy transfer up-conversion rate, which sheds lights on elucidating of the concentration quenching mechanism and mid-infrared luminance behaviors for other investigators. The results reveal that incremental intensity of 2.7 μm emission is caused by the enhancement of ETU1 process and reduction of ETU2 process. Meanwhile, the concentration quenching of 2.7 μm emission happens due to the abrupt change of ETU2 process when reaching the saturation concentration.

## Experimental

The 50 T glasses with molar compositions: 50TeO_2_ - 39RF_2_ (R = Ba, Mg, Zn) - 3NaF - 8 YF_3_ - xErF_3_ (x = 1, 3, 5, 7, 9) were prepared by mixing appropriate quantities of reagent grade raw materials and labeled as 50 T-1Er, 50 T-3Er, 50 T-5Er, 50 T-7Er, 50 T-9Er respectively. Then the 15 g mixture powder batches were melted at 900 °C for 20 min in corundum crucible under air atmosphere. The refined liquids were cast into a pre-heated stainless steel mould and then annealed for 120 min under glass transition temperature. Eventually, the samples were polished and cut to a 1 mm thickness pieces for test. The density indexes of the glasses were measured by the Archimedes method using distilled water as an immersion liquid. The refractive indexes were measured on MOPEL 2010/M Prism Coupler device (Metricon Co. America) at 632.8 nm, 1113 nm and 1539 nm. The thermal property was measured by differential scanning calorimeter (DSC) at the heating rate of 10 °C/min. The temperature of glass transition (T_g_) was 425 °C. The FTIR transmittance spectra of the prepared samples were measured by Tensor 27 FTIR spectrophotometer (Brook Co. Germany). The absorption spectra were recorded by a UV3600 UV/VIS spectrophotometer in the range of 300–1600 nm with a resolution of 0.1 nm. Photoluminescence spectra in the ranges of 1400–1700 nm, 2550–2850 nm and 900–1050 nm were determined by a combined fluorescence lifetime and steady state spectrometer (FLSP920) (Edingburg Co. England), which was detected with a liquidnitrogen cooled PbS detector using an 808 nm LD as an excitation source. The fluorescence lifetimes of the ^4^I_13/2_ state, ^4^I_11/2_ state of Er^3+^ were measured with light pulse of the 808 nm LD and an HP 546800B 100-MHz oscilloscope. For the sake of obtaining comparable results, all the experimental conditions were consistently maintained and carried out at room temperature.

## Results and discussion

### Absorption and infrared transmittance properties

**Figure 1:**
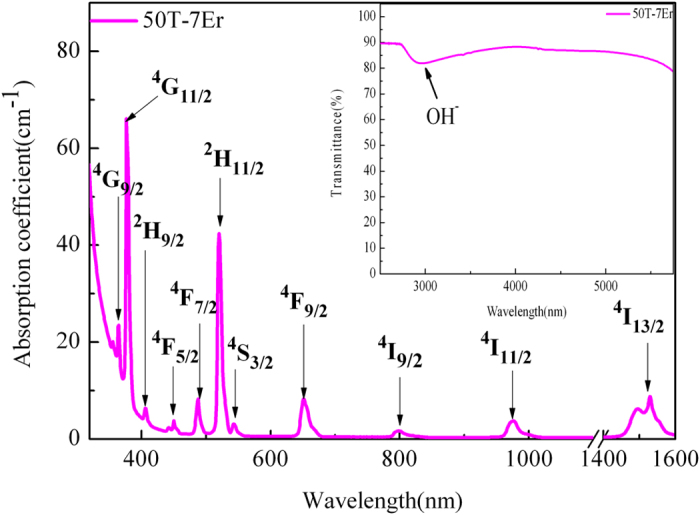
Absorption spectrum of prepared 50 T glass. The inset is sample’s transmittance spectrum.

Figure 1 depicts the room temperature absorption spectrum and transmittance spectrum of 50 T glass doped with 7 mol% Er^3+^. It is easily found that in absorption spectrum the peak positions and shape of each transition of Er^3+^ in present sample are similar to those of other reported glasses^21^. Eleven bands of Er^3+^, centered at 1532 nm, 978 nm, 798 nm, 652 nm, 542 nm, 520 nm, 488 nm, 452 nm, 406 nm, 378 nm and 365 nm, correspond to the transitions originating from the ^4^I_15/2_ ground state to the excited levels ^4^I_13/2_, ^4^I_11/2_, ^4^I_9/2_, ^4^F_9/2_, ^4^S_3/2_, ^2^H_11/2_, ^4^F_7/2_, ^4^F_5/2_, ^2^H_9/2_, ^4^G_11/2_ and ^4^G_9/2_. Other absorption bands are invisible due to the strong intrinsic absorption for present glass. Even though with a relatively weak absorption band at ~808 nm, ^4^I_9/2_ level of Er^3+^ could be resonant with this absorption band. Therefore, this glass could be pump under excitation of 808 nm LD.

As indicated in the inset of Fig.1, the transmittance reaches as high as 90% and extends up to 5.5 μm. The concave existed around 3000 nm is ascribed to typical H_2_O absorption band. It is widely recognized that the OH^−^ group is responsible to the absorption loss and plays as quenching center that reduces 2.7 μm emission intensity and lifetime^14^,^22^. Thus glass with low OH^−^ group concentration is expected for mid-infrared laser application.

The infrared absorption coefficient α_OH_ determined from the transmittance spectrum is commonly utilized to represent OH^−^ group concentration. By using exponential form of Beer’s law^22^:


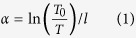


Where l, T_0_ and T represent sample thickness (cm), the maximum transmittance (%) of the glass matrix and the transmittance (%) at 3.0 μm, the Avogadro constant respectively. It was calculated to be 0.674 cm^−1^ in 50 T-7Er glass, which is quite low compared with 1.09 cm^−1^ TY-4 glass^21^ and 0.75 cm^−1^ of TZN glass^23^. Therefore, the host of oxyfluoride tellurite has a considerably low loss band around 3.0 μm, enabling Er^3+^ to emit at ~2.7 μm.

### Judd-Ofelt parameters and radiative properties

Judd-Ofelt (J-O) theory is an effective way to investigate the local structure and boding in the vicinity around rare-earth ions^24^,^25^. The parameters needed for Judd-Ofelt calculation are listed in Table 1. According to the absorption spectrum, three J-O intensity parameters Ω_t_ (t = 2, 4, 6) were calculated. The Ω_2_ = 6.0 ×10^−20^ cm^2^, Ω_4_ = 1.45 × 10^−20^ cm^2^, Ω_6_ = 1.12 × 10^−20^ cm^2^ and the root mean square error δ = 0.34 × 10^−6^ are in well agreement with fluorotellurite glass^26^ and TWPF glass^27^ which indicates the validity of the Judd-Ofelt theory for predicting the spectral intensities of Er^3+^. The Ω_2_ is mostly sensitive to local structure and glass composition. The large value suggests a predominate environment of covalency and asymmetry between rare earth ions and ligands. The Ω_4_, Ω_6_ are related to the rigidity of the host and mostly dependent on bulk properties^28^.

Using the phenomenological J-O parameters, refractive indexes and the equations^29^,^30^, the calculated absorption line strength (S_ed_), calculated predicted spontaneous transition probability (A), radiative lifetime (τ_rad_), and branching ratio (β) of certain optical transitions for 50 T-7Er glass in this study were calculated and shown in Table 2. The A and τ_rad_ of ^4^I_11/2_ → ^4^I_13/2_ transition are crucial parameters for 2.7 μm emission and both relative to the Ω_2_ and refractive index. With large Ω_2_ and refractive index of 50 T-7Er glass, large A (39.97 s^−1^) is obtained and higher than that of Er^3+^-Ho^3+^ co-doped ZBAY glass^31^ (25.11 s^−1^). It is well known that higher spontaneous transition probability provides a better opportunity to obtain laser actions^32^. Therefore this present 50 T-7Er glass could be used as 2.7 μm laser material.

### Mid-infrared fluorescence properties

**Figure 2:**
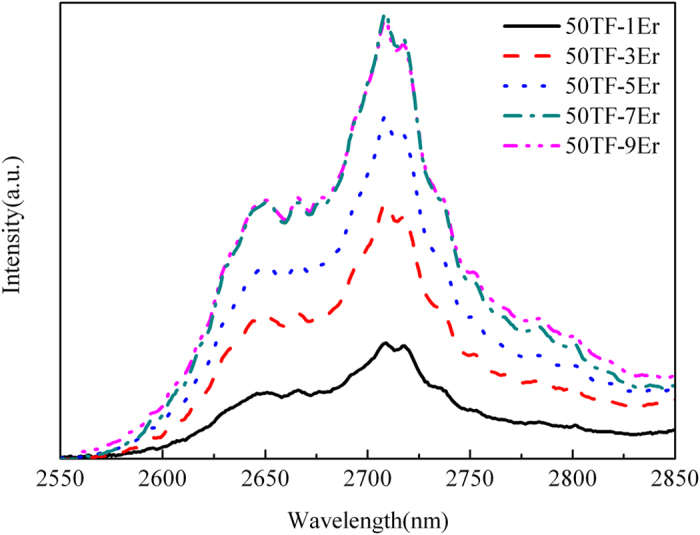
Mid-infrared emission spectra of 50 T glasses doped with 1 mol% to 9 mol% Er^3+^ under 808 nm LD.

The mid-infrared emission spectra of 50 T glasses doped with 1 mol% to 9 mol% Er^3+^ under 808 nm excitation were measured and displayed in the Fig. 2. It can be see that there is intense peak occurring at 2709 nm which attributes to the radiative transition of Er^3+^: ^4^I_11/2_ → ^4^I_13/2_. And the evolution of luminescence intensity is primarily due to the increment of Er^3+^ concentration. The intensity exhibits an increase tendency with the increment of Er^3+^ concentration before reaching to saturation when Er^3+^ is above 7 mol%. Even through the Er^3+^ exceeds 7 mol%, the concentration quenching phenomenon occurs weakly. Therefore, the beneficially high doping Er^3+^ concentration (7 mol%) indicates that the present 50 T host glass is suitable for mid-infrared laser application. It is also clear that there is nearly no change in the line-shape and peak wavelength in all samples.

Important parameters that used to estimate the emission ability of luminescent center for 2.7 μm emission (transition ^4^I_11/2_ → ^4^I_13/2_) include emission cross section (σ_em_), absorption cross section (σ_abs_), effective full width at half-maximum (Δλ_eff_) and product of σ_em_ × Δλ_eff_. The Δλ_eff_ was obtained by the 2.7 μm emission spectra shown in Fig. 2. The σ_em_ could be calculated via Füchtbauer-Ladenburg equation^33^. Moreover, according to the calculated σ_em_ for 2.7 μm emission, the σ_abs_ could be obtained via McCumber theory^34^.


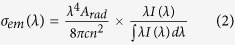



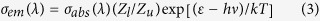


Where A_rad_ is the spontaneous transition probability, I(λ) is the fluorescence spectra intensity, λ is the wavelength, n and c represent the refractive index and the speed of light, Z_l_ and Z_u_ are partition functions of the lower and upper manifolds, respectively.

**Figure 3:**
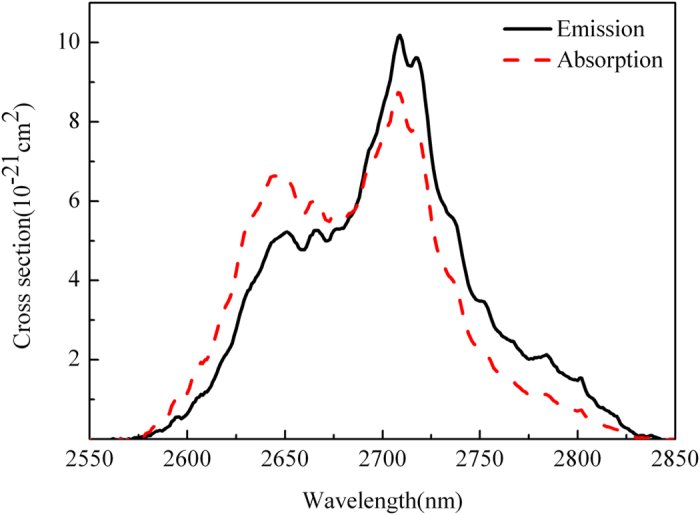
The absorption and emission cross section of 50 T-7Er glass at 2.7 μm.

Figure 3 displays the σ_abs_ (peak value is 8.75×10^−21^ cm^2^) and σ_em_ (peak value is 10.18 × 10^−21^ cm^2^) of 2.7 μm emission for prepared 50 T-7Er glass. In addition, the product of σ_em_ × Δλ_eff_ (945.32 × 10^−28^ cm^3^) was calculated to assess the bandwidth property of the optical amplifier. Table 3 lists the values of these parameters in various Er^3+^ doped glasses for comparison. Results indicate that the developed glass has promising potential for mid-infrared applications.

In order to further evaluate the broadband emission performance of 50 T-7Er glass, it is possible to calculate the net gain coefficient G(λ) according to the following equation^6^:


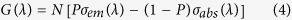


**Figure 4:**
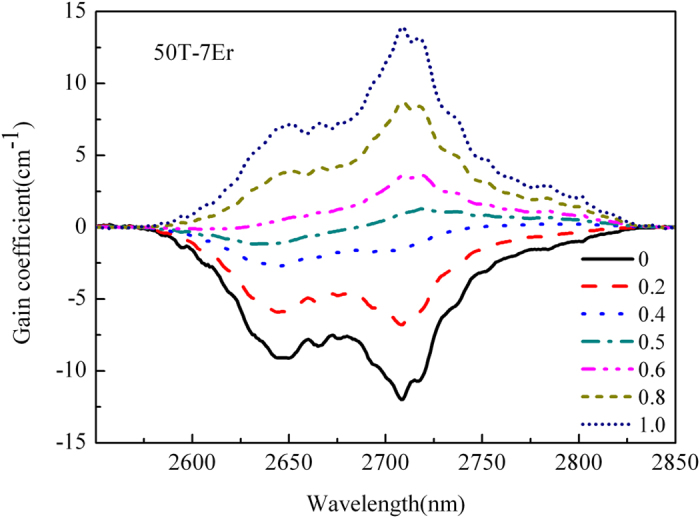
Gain cross section of 50 T-7Er glass.

Where N is the doping concentration of Er^3+^ (13.702 × 10^20^ ions/cm^−3^ for 50 T-7Er glass), P is the population inversion. The 2.7 μm G(λ) with different P value of 50 T-7Er glass as the function of wavelength is depicted in Fig. 4. As shown in the diagram, when the P is 0.5, the G(λ) becomes positive number and is 1.28 cm^−1^. Additionally, the G(λ) has a maximum of 13.95 cm^−1^ when the P is 1. These values are higher than fluorotellurite glass^35^. All of these parameters that mentioned above indicate that the developed glass has promising potential as gain medium for broadband amplifier.

Additionally, the mid-infrared emission spectrum of 50 T-7Er glass under 980 nm excitation was also investigated. The σ_em_ of 2.7 μm emission under 980 nm excitation was calculated to be 10.36 × 10^−21^ cm^2^, which is comparable with that under 808 nm excitation. However, the Δλ_eff_ of 50 T-7Er glass under 980 nm excitation was 80.62 nm, leading to a smaller product of σ_em_ × Δλ_eff_ (835.22 × 10^−28^ cm^3^) than that under 808 nm excitation. Therefore, in present glass system, 808 nm excitation is preferred than 980 nm excitation.

### Energy transfer dynamics and rate equation analysis

**Figure 5:**
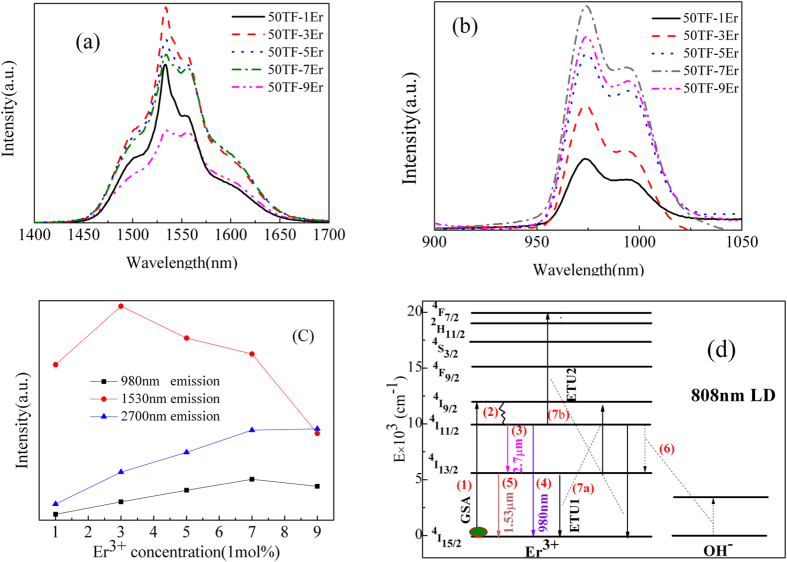
(a) 1.53 μm emission spectra. (b) 980 nm emission spectra. (c) dependence of emission intensity on Er^3+^ doping concentrations. (d) simplified energy level diagram and energy transfer route of Er^3+^.

For the purposes of investigating the energy transfer dynamics, 1.53 μm emission spectra and 980 nm emission spectra with different Er^3+^ concentration were measured under 808 nm excitation and shown in the Fig. 5(a,b) respectively. No band shift but intensity change can be observed in both of the two diagrams due to the variation of Er^3+^ concentration. And according to the Fig. 5(c), which is the dependence of intensity on concentration for 2.7 μm, 1.53 μm and 980 nm emission, the 1.53 μm emission has a quenching concentration of 3 mol% Er^3+^. It is interesting that the 980 nm emission has a similar tendency with 2.7 μm emission. Basing on the luminescent behaviors, the involved energy level diagram is shown in the Fig. 5(d). The energy transfer route should be:

(1). Er^3+^ ions are initially excited by ground state absorption (GSA) of pumping 808 nm energy to ^4^I_9/2_ state from ^4^I_15/2_ state.

(2). Due to the narrow energy gap between ^4^I_9/2_ state and ^4^I_11/2_ state, the electrons of ^4^I_9/2_ state are easily depraved to ^4^I_11/2_ state by multi-phonon relaxation (MPR).

(3). While some electrons in ^4^I_11/2_ state partly relax to ^4^I_13/2_ state with a radiative emission of 2.7 μm by transition of Er^3+^: ^4^I_11/2_ → ^4^I_13/2_ + 2.7 μm photon.

(4). Or partly relax to ^4^I_15/2_ state with a radiative emission of 980 nm by transition of Er^3+^: ^4^I_112_ → ^4^I_15/2_ + 980 nm photon.

(5). Then the populated ^4^I_13/2_ state yields 1.53 μm emission by transition of Er^3+^: ^4^I_13/2_ → ^4^I_15/2_ + 1.53 μm photon.

Additionally, three other processes play an important role in changing the population of Er^3+^: ^4^I_11/2_ state and ^4^I_13/2_ state, therefore, affecting the emission intensity of corresponding states. They are:

(6). Energy transfer process (ET) of Er^3+^: ^4^I_11/2_ → ^4^I_13/2_ + OH^−^.

(7a). Energy transfer up-conversion process (ETU1): Er^3+^: ^4^I_13/2_ + ^4^I_13/2_ → ^4^I_9/2_ + ^4^I_15/2._

(7b). Energy transfer up-conversion process (ETU2): Er^3+^: ^4^I_11/2_ + ^4^I_11/2_ → ^4^F_7/2_ + ^4^I_15/2._

It has known that main factors of affecting 2.7 μm emission intensity are the populations of Er^3+^: ^4^I_11/2_ state and ^4^I_13/2_ state. Since Er^3+^ doping concentration would no obviously alter the OH^−^ content, it should be the energy transfer up-conversion processes that are altered by Er^3+^ doping concentration. With the increment of Er^3+^ doping concentration, energy transfer up-conversion processes are supposed to change correspondingly, resulting in an obvious effect on the populations of ^4^I_11/2_ and ^4^I_13/2_ states. According to the luminescent behaviors, it is thought that when the Er^3+^ concentration is up to 7 mol%, the maximum population gap between the ^4^I_11/2_ state and ^4^I_11/2_ state is obtained. Therefore, the 50 T-7Er glass has strongest 2.7 μm emission intensity among the prepared samples.

**Figure 6:**
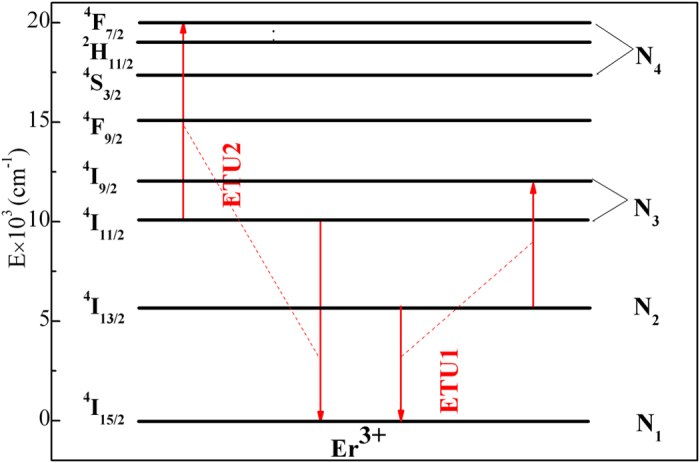
Energy level diagram used for rate equations.

To quantitatively improve the hypothesis, rate equations of the system by using the decay performance are carried out. For the purpose of simplifying calculation, we assumed that four main energy states N_1_, N_2_, N_3_ and N_4_ represent the states of ^4^I_15/2_, ^4^I_13/2_, ^4^I_11/2_ + ^4^I_9/2_, and ^4^S_3/2_ + ^2^H_11/2_ + ^4^F_7/2_ states (as shown in Fig. 6). And after excitation of 808 nm LD, all the Er^3+^ ions distribute among the four energy states, therefore, particle number of Er^3+^ ions equals to population of N_1_ + N_2_ + N_3_ + N_4_. The rate equations for our model are:


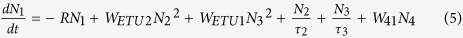



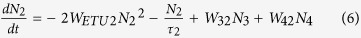



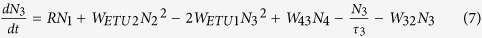



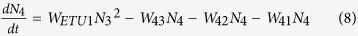


Where I_p_ is the intensity of the pump light and equals to 7.22 W/cm^2^ for 808 nm LD in present work. hν_p_ is the photon energy of the pump radiation. R = σ_abs_(I_p_/hν_p_) is the pump rate (s^−1^). W_ij_ is the total energy transfer rate from upper i state to lower j state (including radiative and non-radiative transfer processes). W_ETU_ is the energy transfer up-conversion rate. τ_2_ is the measured lifetime of ^4^I_13/2_ state under 808 nm LD excitation a. τ_3_ is the measured lifetime of ^4^I_11/2_ state under 808 nm LD excitation. The details of measured lifetimes are displayed in Table 4.

Take the ETU1 process of ^4^I_13/2_ state as example. Yamauchi assumed that no other processes influence the population of state N_2_ after the pump power is turn off (R = 0) and Eqs.(6) could be simplified as^36^:


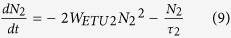


Then solve Eqs.(9), we can obtain the following function expression:


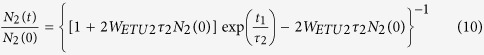


Where N_2_(0) is population of ^4^I_13/2_ state after the pump power is turn off. Then by solving the Eqs.(10) in steady-state condition, we can get the equation:


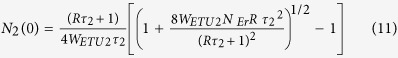


**Figure 7:**
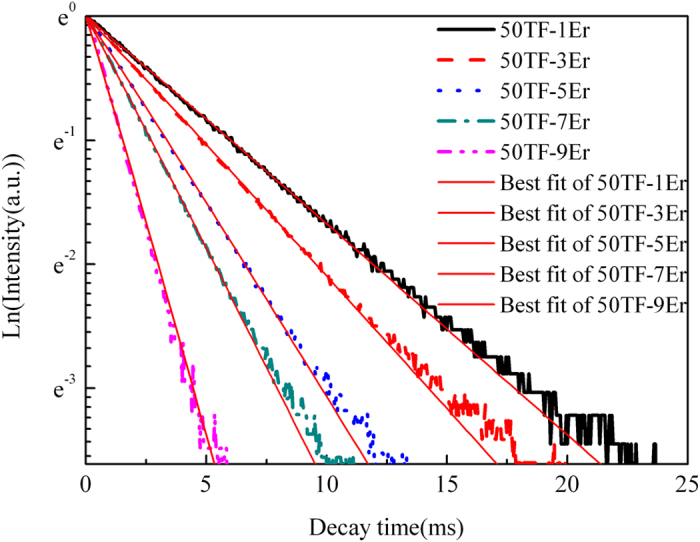
Decay curves of 1.53 μm emission in different Er^3+^ doping concentration glasses excited by 808 nm LD.

The parameters energy transfer up-conversion rate of ^4^I_13/2_ state (W_ETU1_) could be obtained by fitting Eq.(11) to the normalized fluorescence decay curves of ^4^I_13/2_ state. The results are shown in Fig. 7. Seen from the figure, the fitting curves match well with the measured lifetimes, illustrating the validity of fitting procedure. The fitting procedure of ETU2 process is similar with that of ETU1 process, therefore, we would not discuss in detailed.

**Figure 8:**
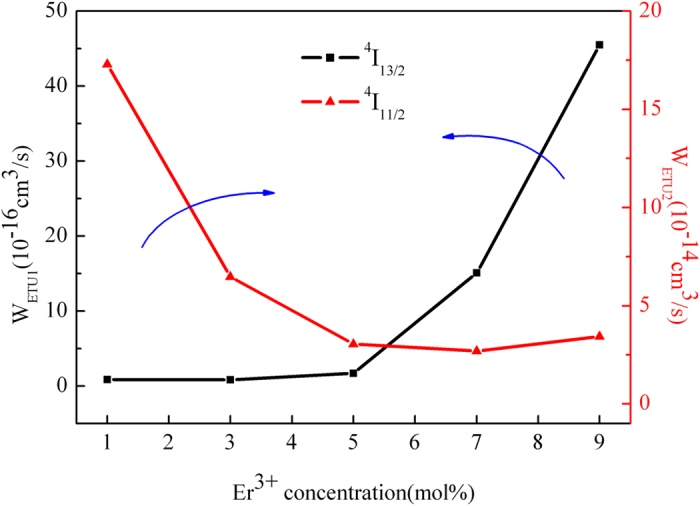
Fitting results of parameters W_ETU_ with different Er^3+^ doping concentration.

The dependence of W_ETU_ on Er^3+^ concentration are shown as the Fig. 8. It is obvious that the W_ETU1_ of ^4^I_13/2_ state substantially increases with the increment of Er^3+^ concentration. The increase of W_ETU1_ is advantageous to deplete the ions in ^4^I_13/2_ state by transferring energy to adjacent state. In parallel, the W_ETU2_ of ^4^I_11/2_ state reduces with the increment of Er^3+^ concentration. The lower W_ETU2_ makes more ions aggregated in ^4^I_11/2_ state. Therefore the population inversion between the ^4^I_11/2_ and ^4^I_13/2_ state is deeply improved and the 2.7 μm emission is enhanced with the increment of Er^3+^ concentration.

## Conclusion

In summary, a detailed investigation of Er^3+^ doped oxyfluoride tellurite glasses with molar compositions of 50TeO_2_ - 39RF_2_ (R = Ba, Mg, Zn) - 3NaF - 8 YF_3_ - xErF_3_ (x = 1, 3, 5, 7, 9) is carried out. Judd-Ofelt intensity parameters and radiative properties of 50 T-7Er glass are calculated and discussed. Emission spectra consisted of 2.7 μm, 1.53 μm and 980 nm emission under 808 nm excitation are investigated. The results indicate that 50 T-7Er glass is a potential candidate for mid-infrared laser application. A possible energy transfer mechanism is proposed basing on the luminescence behaviors and the rate equation analysis is made to quantitatively prove it. With the increment of Er^3+^ concentration, the energy transfer up-conversion rate W_ETU1_ of ^4^I_13/2_ state increases while the energy transfer up-conversion rate W_ETU2_ of ^4^I_112_ state reduces, leading a increment of population inversion between the two states. Therefore, the 2.7 μm emission is enhanced with the increment of Er^3+^ concentration.

## Additional Information

**How to cite this article**: Chen, F. *et al.* Investigation of mid-infrared emission characteristics and energy transfer dynamics in Er^3+^ doped oxyfluoride tellurite glass. *Sci. Rep.* **5**, 10676; doi: 10.1038/srep10676 (2015).

## Figures and Tables

**Table 1:** Physical properties of the 50 T-7Er glass.

**Properties**	**50T-7Er**
Density, d (g·cm^−3^)	5.069
Average molar weight, (g·mol^−1^)	155.89
Er^3+^ concentration. C (10^20^ cm^−3^)	13.702
Refractive index, n at 632.8 nm	1.7378
Refractive index, n at 1311 nm	1.7162
Refractive index, n at 1539 nm	1.7130
Temperature of glass transition, T_g_ (°C)	425

**Table 2:** The calculated absorption line strength (S_ed_), calculated predicted spontaneous transition probability (A), radiative lifetime (τ_rad_), and branching ratio (β) of 50 T-7Er glass.

**Transition**		**λ(nm)**	**S_ed_(10^−20^ cm^2^)**	**A(s^−1^)**	β**(%)**	**τ_rad_(ms)**
^4^I_13/2_→	^4^I_15/2_	1530	1.89	187.29	100	5.34
^4^I_11/2_→	^4^I_15/2_	977	0.61	192.52	82.81	4.30
→	^4^I_13/2_	2703	1.66	39.97	17.19	
^4^I_9/2_→	^4^I_15/2_	798	0.26	181.55	73.17	4.03
→	^4^I_13/2_	1667.95	0.82	62.16	25.06	
→	^4^I_11/2_	4355.56	0.26	4.40	1.77	
^4^F_9/2_→	^4^I_15/2_	651	1.29	1649.69	71.21	0.43
→	^4^I_13/2_	1133.14	2.36	571.60	24.67	
→	^4^I_11/2_	1951.00	1.88	88.90	3.84	
→	^4^I_9/2_	3534	0.81	6.44	0.28	
^4^S_3/2_→	^4^I_15/2_	543	0.25	1360.42	66.47	0.49
→	^4^I_13/2_	841.73	0.39	571.86	27.94	
→	^4^I_11/2_	1222.34	0.89	42.79	2.09	
→	^4^I_9/2_	1699.27	0.40	71.52	3.49	

**Table 3:** σ_abs_, σ_em_ and the products of σ_em _× Δλ_eff_ of 50T-7Er glass compared with other glasses.

	**50 T-7Er**	**FPY1**^37^	**GGL**^38^	**FP**^8^	**5T-ZBLAN**^39^
σ_abs_(10^−21^ cm^2^)	8.75	--	6.17	--	--
σ_em_(10^−21^ cm^2^)	10.18	8.17	7.78	6.57	6.32
Δλ_eff_ (nm)	92.86	93.5	54.5	~80	100
σ_em_×Δλ_eff_(10^−28^ cm^3^)	945.32	763.895	424.01	~525.6	632

**Table 4:** Lifetimes of ^4^I_13/2_ state (τ_2_) and ^4^I_11/2_ state (τ_3_) excited by 808 nm LD with different Er^3+^ doping concentrations. The Adj.R-square stands for the fitting precision.

	**1 mol % Er^3+^**	**3 mol % Er^3+^**	**5 mol % Er^3+^**	**7 mol % Er^3+^**	**9 mol % Er^3+^**
τ_2_(ms)	5.85	4.65	3.16	2.58	1.44
Adj.R-square	0.9997	0.9998	0.9997	0.9997	0.9988
τ_3_(ms)	0.324	0.301	0.279	0.273	0.246
Adj.R-square	0.9991	0.9987	0.9987	0.9988	0.9983
